# Growth of HIV-Exposed Uninfected Infants in the First 6 Months of Life in South Africa: The IeDEA-SA Collaboration

**DOI:** 10.1371/journal.pone.0151762

**Published:** 2016-04-06

**Authors:** Erna Morden, Karl-Günter Technau, Janet Giddy, Nicola Maxwell, Olivia Keiser, Mary-Ann Davies

**Affiliations:** 1 School of Public Health and Family Medicine, University of Cape Town, Cape Town, South Africa; 2 Rahima Moosa Mother and Child Hospital and University of the Witwatersrand, Johannesburg, South Africa; 3 McCord Hospital, Durban, South Africa; 4 Institute of Social and Preventive Medicine, University of Bern, Bern, Switzerland; George Washington University, UNITED STATES

## Abstract

**Background:**

HIV-exposed uninfected (HEU) infants are a growing population in sub-Saharan Africa especially with the increasing coverage of more effective prevention of mother-to-child transmission (PMTCT) antiretroviral therapy regimens. This study describes the characteristics of South African HEU infants, investigates factors impacting birth weight and assesses their growth within the first 28 weeks of life.

**Methods:**

This is a retrospective cohort based on routine clinical data from two South African PMTCT programmes. Data were collected between 2007 and 2013. Linear regression assessed factors affecting birth weight-for-age z-scores (WAZ) while growth (longitudinal WAZ) was assessed using mixed effects models.

**Results:**

We assessed the growth of 2621 HEU infants (median birth WAZ was -0.65 (IQR -1.46; 0.0) and 51% were male). The feeding modalities practised were as follows: 0.5% exclusive breastfeeding, 7.9% breastfeeding with unknown exclusivity, 0.08% mixed breastfeeding and 89.2% formula feeding. Mothers with CD4 <200 cells/μl delivered infants with a lower birth WAZ (adjusted ß -0.253 [95% CI -0.043; -0.072], p = 0.006) compared to mothers with aCD4 ≥500 cells/μl. Similarly, mothers who did not receive antiretroviral drugs delivered infants with a lower birth WAZ (adjusted ß -0.39 [95% CI -0.67; -0.11], p = 0.007) compared to mothers who received antenatal antiretrovirals. Infants with a birth weight <2 500g (ß 0.070 [95% CI 0.061; 0.078], p <0.0001) experienced faster growth within the first 28 weeks of life compared to infants with a birth weight ≥2 500g. Infants with any breastfeeding exposure experienced slower longitudinal growth compared to formula fed infants (adjusted ß -0.012 [95% CI 0.021; -0.003], p = 0.011).

**Conclusion:**

Less severe maternal disease and the use of antiretrovirals positively impacts birth weight in this cohort of South African HEU infants. Formula feeding was common with breastfed infants experiencing marginally slower longitudinal growth.

## Introduction

HIV-exposed uninfected (HEU) infants are a growing population in sub-Saharan Africa, particularly with increasing coverage of more effective prevention of mother-to-child transmission (PMTCT) regimens such as option B/B+, antiretroviral therapy (ART) for all pregnant and breastfeeding women either until cessation of breastfeeding or lifelong [1]. In 2010, an evaluation of the South African PMTCT programme, where antenatal HIV prevalence was 32%, found the overall early HIV transmission rate (4–8 weeks postpartum) to be 3.5% [2]—indicating a large number of exposed but uninfected infants. More recently, antenatal HIV prevalence is 29.5% in South Africa and both effectiveness and coverage of PMTCT have improved [3].

Breastfeeding is known to transmit HIV, however its importance for infant nutritional status, growth [4–8] and protection against morbidity [9] and mortality [10–13] is also well documented. In South Africa particularly, mixed feeding, defined by the World Health Organization (WHO) as a combination of breast milk and/or infant formula, other liquids and solids [14,15], is common. In an evaluation of the South African PMTCT programme, 53% of HIV-infected mothers breastfed their infants, of whom 42% practised exclusive breastfeeding (EBF) at 3 weeks of age. By 12 weeks of age, however, only 18% of HIV-infected mothers who breastfed their infants were practising EBF. Among HIV-infected mothers who practised mixed breastfeeding (MBF) at 12 weeks of age, 48% and 52% of infants were partially (breast milk and non-nutritive and nutritive solids and liquids) and predominantly (breast milk and non-nutritive liquids) breastfed respectively [14]. Similarly, Coutsoudis et al found 57% of infants were mixed fed in a study conducted early in the HIV/AIDS pandemic, prior to the PMTCT programme [9], thus mixed feeding is widely practised in South Africa.

There have been conflicting findings with respect to the effect of feeding on growth. McGrath et al found no differences in the rate of growth between ever breastfed and formula fed infants, with WAZ increasing in both groups during the first 6 months after which they declined [8]. However, Malawian studies found that not breastfeeding was associated with both an increased risk of being underweight (WAZ <-2) as well as having lower weight-, length- and weight-for-length z-scores compared to infants who were breastfed [6,7]. The Zambia Exclusive Breastfeeding Study found that among all HEU infants there was a decline in WAZ between 4.5 and 15 months, but that in infants who had continued breastfeeding at 4 months, the decline was lessened [16]. The early introduction of cow’s milk (≤ 6 weeks of age) was also found to have a negative impact on growth [17]. The effect of feeding modality on growth may in part be context-dependent, and affected by the extent to which the affordable, feasible, accessible, safe and sustainable (AFASS) criteria for replacement feeding are met [14]. Longitudinal growth is not only affected by feeding but by birth weight and maternal factors too. Infants with a higher birth weight have greater postnatal weight-for-age (WAZ) over time compared to low birth weight (LBW) infants (<2 500g) although postnatal growth rate in the first year of life is faster in LBW infants [4,5,8,16,17]. There have been conflicting findings with respect to the effect of feeding on growth. Maternal health also impacts child growth: infants of mothers with advanced disease (high viral load, ≥100 000 copies/ml, or low CD4 cell count, ≤350 cells/μl) were found to have slower growth [5,16,17].

Many previous studies of growth in HEU were conducted outside South Africa, where breastfeeding practices and access to replacement feeding may be different, and prior to widespread availability of effective PMTCT regimens. The aim of this analysis was therefore to assess birth weight and growth within the first 6 months of life in HEU infants from two PMTCT cohorts in South Africa and examine the impact of maternal factors, including disease severity and the use of antiretrovirals, and feeding modality.

## Methods

### Study design, setting and participants

This was a retrospective cohort study based on routine data provided to the International Epidemiologic Databases to Evaluate AIDS, Southern Africa (IeDEA-SA, www.iedea-sa.org) collaboration. The IeDEA-SA cohort has been previously described [18,19], although this is the first analysis of HEU infants. Two South African sites were included in this analysis namely McCord Hospital (MH) in KwaZulu-Natal Province and Rahima Moosa Mother and Child Hospital (RMMCH), Gauteng Province. MH was a public-not-for-profit programme where a small patient co-payment was required at each visit, while RMMCH is a public hospital where care is provided at no cost to pregnant women and children ≤6 years old. Both facilities provided primary and secondary care. We included infants born from 2007–2013. Growth monitoring and promotion was provided based on standard practices at each facility. In South Africa, 2008 guidelines on infant feeding advocated HIV-infected mothers exclusively breastfeed their infants unless replacement feeding met the AFASS criteria, in which case free infant formula was provided for 6 months [20,21]. The 2010 PMTCT guidelines recommended the following antiretroviral regimens: women were eligible for lifelong ART (tenofovir (TDF) + lamivudine/emtricitabine (FTC) + nevirapine (NVP)) if they had a CD4 ≤350 cells/μl or WHO clinical stage 3/4. Women not eligible for ART (CD4 >350 cells/μl or WHO clinical stage 1/2) received zidovudine (ZDV) from 14 week gestation followed by single dose NVP and ZDV during labour and TDF + FTC after delivery [22]. Prior to 2010, women at RMMCH received single dose nevirapine (sdNVP) at delivery only, while at MH ZDV from 14 weeks gestation plus sdNVP at delivery was used in women ineligible for triple ART (CD4 ≥200 cells/μl). In 2011, the Tshwane declaration of support for breastfeeding was signed, ending the provision of infant formula for PMTCT in state facilities, like RMMCH [23]. MH continued the provision of infant formulae.

We included HIV-exposed uninfected infants with at least birth weight and one subsequent weight measurement within the first 28 weeks of life. The following maternal information was included for the mother-infant pair: age, parity, antenatal CD4 count and antenatal anteretroviral use. We excluded infants diagnosed as HIV-infected (n = 126), most were diagnosed (97%) before 3 months of age (8.7% diagnosed by 1 month, 89% between 1–3 months, 1.6% between 3–6 months and 0.8% after 12 months of age).

### Ethics statement

The IeDEA Southern Africa Collaboration has been approved by both the University of Cape Town and University of Bern Human Research Ethics Committees. Rahima Moosa Mother and Child Hospital and McCord Hospital have local institutional ethics approval to contribute data to the IeDEA-SA analyses from the University of Witwatersrand and University of Cape Town, respectively. Participating sites obtained informed consent from all subjects. The data were anonymised and de-identified prior to analyses.

### Outcomes

The primary outcome was postnatal growth within the first 28 weeks of life which was assessed using WAZ only. We did not assess growth using length-for-age or weight-for-length as length measurements were not available on all infants at all time points. Factors influencing birth weight were also assessed as birth weight impacts longitudinal postnatal growth.

When assessing postnatal growth, birth weight was considered the baseline and follow-up visits with recorded weights were used to assess growth. WAZ was calculated using the WHO Child growth standards (igrowup, version 3.2.2 2011) package for Stata^®^. All statistical analyses were done using Stata^®^ (StataCorp. 2011. Stata Statistical Software: Release 12. College Station, TX: StataCorp LP).

### Variables

We collected data on the following maternal factors: parity (number of previous live births) for the index pregnancy (categorised as 0, 1 and ≥2); maternal age [<18 years (younger), 18–35 years and >35 years (older)]; antenatal CD4 cell count (<200, 200–500 and ≥500 cells/μl) and maternal antiretrovirals grouped into any antiretrovirals (comprising unknown regimen, nevirapine (NVP) only, dual therapy and triple therapy), no antiretrovirals and missing antiretroviral information. Where maternal antiretroviral regimen was changed during the pregnancy, we included the antenatal regimen closest to the delivery date.

Infant variables collected included the following: sex, gestational age (based on palpation or date of last menstrual period) at delivery (term (≥ 37 weeks), preterm and unknown gestational age), birth weight (low (<2 500g) or normal (≥2 500g). Infant feeding, as it occurred over 28 weeks, was categorised into formula feeding (FF i.e. never breastfed), unknown feeding and any breastfeeding (BF). Any BF comprised EBF, BF with unknown exclusivity and MBF.

### Analysis

Baseline characteristics between sites were compared using Wilcoxon rank-sum test for continuous variables and Chi-squared or Fisher’s exact tests for categorical variables.

We first assessed factors affecting birth weight using univariate and multivariate linear regression. We included gestational age, infant sex, maternal age and parity, and cohort in the model *a priori* and then examined the effect of maternal HIV disease severity and treatment (antenatal CD4 cell count and the use of antiretrovirals) adjusted for the *a priori* variables. Univariate and multivariate analyses of the association between demographic, feeding and maternal factors affecting longitudinal postnatal growth were examined using mixed effects models with maximum likelihood estimation. *A priori* inclusions were similar to the linear regression model investigating birth weight outcomes. Feeding modality, infant age (continuous variable, in weeks) and birth weight were additional *a priori* inclusions. The model was then adjusted for maternal age, parity, antenatal CD4 count and antiretroviral use. As growth is time dependent, interaction terms with infant age in weeks were included for all covariates. Covariates (including interaction terms) were included if the p-value was <0.1. As birth weight was a major determinant of postnatal growth, we conducted a sensitivity analysis limited to children with normal birth weight to examine the factors associated with growth in this subset of children. Owing to the nature of routine clinical data, not all variables were assessed at every visit or within the time period of this analysis. Cohort was adjusted for in all analyses.

## Results

**Fig 1:**
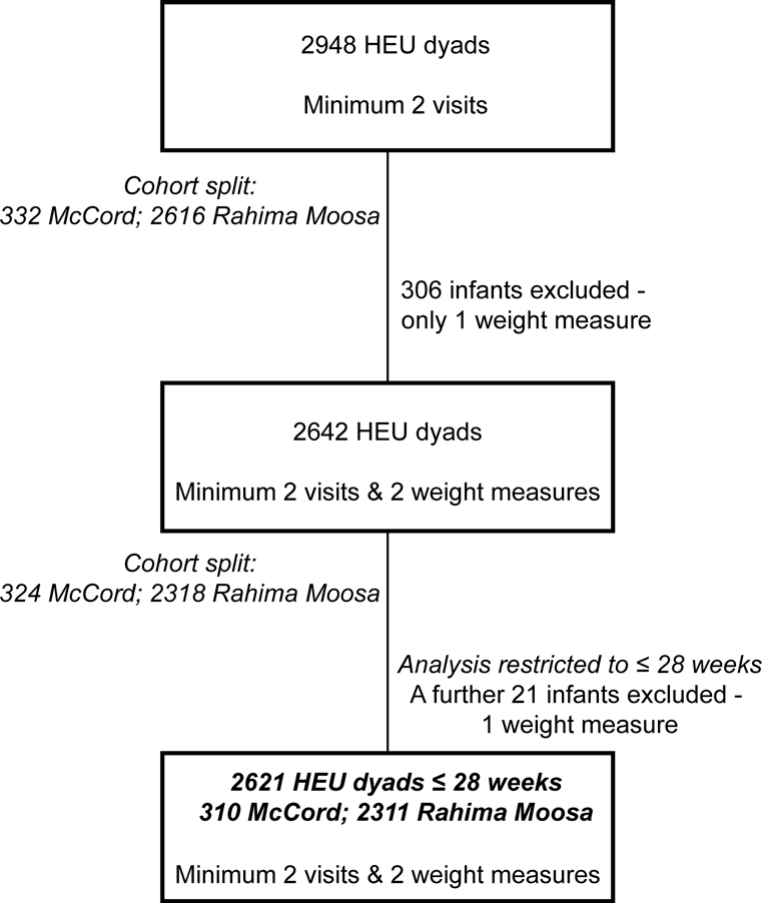
HEU profile.

There were 2948 dyads with a minimum of 2 visits, of these 327 (11.1%) were excluded as they did not have at least one visit after birth within the first 28 weeks of life. The HEU profile of the dyads included in the analyses is detailed in Fig 1.

### Baseline maternal and infant characteristics

The median age at visits within the first 28 weeks was 2.17 months (IQR 1.48; 2.53 months). The majority of the infants in this analysis were from RMMCH (Table 1). Overall, 83% of infants had a normal birth weight (>2 500g) and 51% were male. The overall median birth WAZ was -0.65 (IQR -1.46; 0.00); lower at RMMCH (-0.68, IQR -1.49; -0.04) compared to MH (-0.52, IQR -1.23; 0.31). Gestational age at delivery was only available for 70% of infants (median 39 weeks; IQR 37; 40). Formula feeding was most commonly practised overall (89.2%), with similarities between sites; 89.1% and 90% of infants at RMMCH and MH were formula fed respectively. Few infants were mixed fed at MH (0.65%) with no mixed feeding recorded at RMMCH. Maternal characteristics are detailed in Table 2. The median maternal age at delivery was similar between the two sites, with most mothers between 25–35 years old. At MH, where maternal regimen was recorded, 74.8% were on triple therapy (mostly non-nucleo(s)tide reverse transcriptase inhibitors (NNRTI) based), 18.4% on dual therapy, 0.7% NVP only and 3.6% did not receive antiretrovirals. For most mothers at RMMCH who received antiretrovirals for PMTCT, the regimen was not recorded (69.9%). A further 15.5% received NVP only, 0.43% triple therapy, 2.9% dual therapy, and 8.5% did not receive antiretrovirals. For mothers who had antenatal CD4 cell count recorded, 33.6% had a CD4 cell count between 200–500 cells/μl.

**Table 1:** HEU infant characteristics.

	Characteristics	McCord				Rahima Moosa				Overall			
		n	% missing	median	IQR	n	% missing	median	IQR	n	median	IQR	p-value
	Infants (n)	310				2311				2621			
	% male	53.9%				50.6%				51.0%			0.277^#^
	normal (>2500g) birth weight (n)	279		90%		1892		81.9%		2171	82.8%		
*anthropometry*	*birth z-scores*												
	birth weight	310	0%	-0.52	-1.23; 0.31	2311	0%	-0.68	-1.49; -0.04	2621	-0.65	-1.46; 0.00	0.0005*
	Males	167		-0.35	-1.09; 0.31	1169		-0.64	-1.42; 0.04	1336	-0.6	-1.42; 0.065	
	Females	143		-0.52	-1.47; 0.15	1142		-0.75	-1.56; -0.08	1285	-0.73	-1.52; -0.07	
	birth length	302	2.6%	0.06	-0.62; 1.53	2184	5.50%	0.06	-1.15; 1.12	2486	0.06	-1.0; 1.12	0.0059*
	Males	161		0.06	-0.47; 1.12	1109		0.06	-1.0; 1.12	1269	0.06	-1.0; 1.12	
	Females	141		0.46	-0.62; 1.53	1076		-0.08	-1.15; 0.99	1217	-0.08	-1.15; 0.99	
	birth head circumference	301	2.9%	0.42	-0.36; 1.21	2178	5.76%	0.1	-0.74; 0.95	2479	0.1	-0.74; 0.95	0.0001*
	Males	162		0.42	-0.36; 1.21	1099		-0.36	-1.15; 1.21	1261	0.42	-1.15; 1.21	
	Females	139		0.1	-0.74; 0.95	1079		0.1	-0.74; 0.95	1218	0.1	-0.74; 0.95	
*delivery*	*delivery variables*												
	gestational age (weeks)	156	49.7%	38	38; 39	1674	27.56%	39	37; 41	1830	39	37; 40	0.0411*
	1 min apgar	288	7.1%	8	7;8	1723	25.44%	9	9;9	2011	9	8;9	<0.0001*
	5 min apgar	288	7.1%	9	9;9	1689	26.91%	10	9;10	1977	10	9; 10	<0.0001*
*feeding*	*Infant feeding (at birth/1*^*st*^ *visit)*												<0.0001^#^
	Exclusive breastfeeding	10		3.2%		3		0.1%		13	0.5%		
	Breastfeeding, exclusivity unknown	1		0.3%		206		8.9%		207	7.9%		
	Mixed feeding	2		0.7%		-				2	0.1%		
	Formula feeding	279		90.0%		2059		89.1%		2338	89.2%		
	Unknown feeding	18		5.8%		43		1.9%		61	2.3%		
*visits*	visits per patient (n = total obs)	1548		3	2;5	6600		2	1;3	8148	2	1;3	<0.0001*

IQR: inter-quartile range; NVP: nevirapine; AZT: zidovudine; NNRTI: non-nucleot(s)ide reverse transcriptase inhibitors; PI: protease inhibitors

*Wilcoxon rank-sum test

#Chi-squared/Fisher’s exact test

**Table 2:** HEU maternal characteristics.

Characteristics		McCord			Rahima Moosa			Overall			
		n	median	IQR	n	median	IQR	n	median	IQR	p-value
*Age*	delivery age (years)	310	29.81	25.63; 33.62	2303	29.57	25.54; 33.56	2613	29.61	25.57; 33.56	0.7995*
	*age categories*										0.473^#^
	missing	-	-		8	0.4%		8	0.3%		
	younger mothers	63	20.3%		500	21.6%		563	21.5%		
	25–35 years	191	61.6%		1369	59.2%		1560	59.5%		
	older mothers	56	18.1%		434	18.8%		490	18.7%		
*Parity*	*parity*										<0.0001^#^
	missing	165	53.2%		126	5.4%		291	11.1%		
	0	48	15.5%		481	20.8%		529	20.2%		
	1	62	20.0%		842	36.4%		904	34.5%		
	2+	35	11.3%		862	37.3%		897	34.2%		
*CD4 cells/*μ*l*	*CD4 cells/*μ*l during pregnancy*	229	316	207; 471	1330	362	240; 501	1559	357	236; 500	0.0140*
	*Categorised CD4 cell count*										<0.0001^#^^‡^
	missing	81	26.1%		981	42.5%		1062	40.5%		
	<100	10	3.2%		56	2.4%		66	2.5%		
	100–200	38	12.3%		176	7.6%		214	8.2%		
	200–350	81	26.1%		397	17.2%		478	18.2%		
	350–500	47	15.2%		356	15.4%		403	15.4%		
	≥500	53	17.1%		345	14.9%		398	15.2%		
*Antiretrovirals*	*Antiretroviral (ARV) summary*										0.002^#^^§^
	no drugs	11	3.6%		196	8.5%		207	7.90%		
	NVP only	2	0.7%		357	15.5%		359	13.7%		
	AZT only or AZT-NVP combination	48	15.5%		66	2.9%		114	4.4%		
	other dual therapy	9	2.9%		-			9	0.3%		
	NNRTI regimen	198	63.9%		10	0.4%		208	7.9%		
	PI regimen	34	11.0%		-			34	1.3%		
	Received ARVs, regimen unknown	4	1.3%		1615	69.9%		1619	61.8%		
	Missing	4	1.3%		67	2.9%		71	2.7%		

IQR: inter-quartile range; ARVs: antiretroviral drugs; NVP: nevirapine; AZT: zidovudine; NNRTI: non-nucleot(s)ide reverse transcriptase inhibitors; PI: protease inhibitors

*Wilcoxon rank-sum test

#Chi-squared/Fisher’s exact test

‡Included all categories of CD4, including missing

§Any ARVs, no ARVs and missing ARVs were compared

### Factors affecting birth weight

The models investigating the factors affecting birth weight are found in Tables 3 and 4. Infants with a gestational age <37 weeks had a lower birth WAZ compared to term infants (p <0.0001). Females had a lower birth WAZ compared to males (p = 0.005), in agreement with previous studies [7,12,13,24–26]. When looking at maternal factors, mothers who received antiretrovirals, irrespective of regimen, delivered infants with a higher birth WAZ. In comparison to women with a CD4 cell count >500cells/μl, those with lower CD4 cells counts delivered infants with a lower birth WAZ but this was only significant for mothers with a CD4 <200cells/μl (p = 0.006). Older mothers (>35 years) gave birth to infants with a significantly lower birth WAZ compared to mothers 25–35 years old (p = 0.007).

**Table 3:** Linear regression weight-for-age z-scores including parity and maternal CD4, birth weight; n = 1397.

	*unadjusted*			*Adjusted*		
*variables*	unadjusted ß	95% CI	p-value	adjusted ß	95% CI	p-value
Term ≥ 37 weeks	0			0		
Premature^†^	-0.865	-1.03; -0.700	*	-0.829	-0.995; -0.663	*
Unknown GA	-0.056	-0.204; 0.092	0.461	-0.028	-0.176; 0.0120	0.713
Any ARVs	0			0		
No ARVs	-0.490	-0.781; -0.199	0.001	-0.387	-0.668; -0.106	0.007
ARVs missing information	-0.326	-0.876; 0.225	0.246	-0.247	-0.776; 0.281	0.359
25–35 years^¶^	0			0		
Young mother	-0.010	-0.165; 0.144	0.895	0.036	-0.123; 0.194	0.657
Older mother	-0.217	-0.378; -0.056	0.008	-0.221	-0.381; -0.061	0.007
Male sex	0			0		
Female sex	-0.200	-0.321; -0.079	0.001	-0.164	-0.280; -0.049	0.005
CD4 ≥500	0.000			0		
CD4 < 200	-0.309	-0.497; -0.122	0.001	-0.253	-0.0434; -0.072	0.006
200 < CD4 < 500	-0.074	-0.217; 0.068	0.305	-0.091	-0.228; 0.045	0.19
parity = 0	0			0		
parity = 1	0.212	0.053; 0.372	0.009	0.263	0.105; 0.420	0.001
parity ≥ 2	0.084	-0.075; 0.243	0.303	0.211	0.042; 0.381	0.015

GA: gestational age

†Gestational age <37 weeks

¶Maternal age

*p<0.0001

**Table 4:** Linear regression weight-for-age z-scores, birth weight; n = 2621.

		*unadjusted*			*adjusted*	
*variables*	unadjusted ß	95% CI	p-value	adjusted ß	95% CI	p-value
Term ≥ 37 weeks	0			0		
Premature^†^	-0.926	-1.07; -0.784	*	-0.865	-1.007; -0.724	*
Unknown GA	-0.405	-0.515; -0.294	*	-0.398	-0.510; -0.287	*
Any ARVs	0			0		
No ARVs	-0.492	-0.678; -0.306	*	-0.375	-0.556; -0.194	*
ARVs missing information	-0.507	-0.816; -0.198	0.001	-0.376	-0.677; -0.074	0.015
25–35 years^¶^	0			0		
Young mother	-0.025	-0.151; 0.102	0.700	-0.008	-0.130; 0.114	0.902
Older mother	-0.299	-0.432; -0.166	*	-0.251	-0.379; -0.122	*
Unknown age^¶^	-0.978	-1.89; -0.065	0.036	-0.592	-1.481; 0.296	0.191
Male sex	0			0		
Female sex	-0.134	-0.234; -0.033	0.009	-0.110	-0.207; -0.013	0.027

GA: gestational age

†Gestational age <37 weeks

¶Maternal age

*p<0.0001

As a sensitivity analysis these factors (except maternal CD4 and parity where there was substantial missing data) were assessed for the entire HEU cohort, not limited to dyads with complete data on all variables, (Table 4), and they remained consistent, albeit slightly greater in magnitude.

### Effects on growth

The main outcome of this analysis is longitudinal growth of HEU infants. The main model is presented in Table 5, left side. This model includes parity and thus excludes all dyads missing information on parity (11.10% overall). The second model, right side of Table 5, shows the magnitude of effects among all infants, excluding any variables with missing information. Lastly, in S1 Table, the model including maternal HIV-related variables (CD4 and ARVs) is shown. Dyads with missing maternal age (0.31% overall), maternal CD4 cell count (40.52% overall) and parity are excluded. In the reference group of HEU infants (model including parity, Table 3), for male infants of nulliparous women with a birth weight >2 500g born at RMMCH and who received infant formula, WAZ increased from birth through 28 weeks of age (adjusted ß 0.019, p-value for interaction <0.0001).

**Table 5:** Longitudinal linear regression^†^ weight-for-age z-scores 0–28 weeks.

	*Model including parity; n = 2328*						*Model including all infants*, *n = 2621*					
	*unadjusted*			*Adjusted*			*unadjusted*			*adjusted*		
*variables*	unadjusted ß	95% CI	p-value	adjusted ß	95% CI	p-value	unadjusted ß	95% CI	p-value	adjusted ß	95% CI	p-value
formula feeding	0			0			0			0		
any breastfeeding	0.059	-0.115; 0.232	0.506	0.071	-0.063; 0.206	0.298	0.123	-0.054; 0.300	0.173	0.085	-0.048; 0.218	0.211
breastfeeding x age	-0.014	-0.024; -0.005	0.003	-0.012	-0.021; -0.003	0.011	-0.017	-0.026; -0.009	*	-0.012	-0.020; -0.003	0.007
unknown feeding	-0.062	-0.039; 0.265	0.712	-0.007	-0.260; 0.247	0.958	-0.116	-0.441; 0.210	0.485	-0.016	-0.028; -0.003	0.017
unknown feeding x age	-0.006	-0.021; 0.008	0.405	-0.010	-0.024; 0.004	0.173	-0.011	-0.025; 0.002	0.096	-0.028	-0.273; 0.216	0.821
birth weight ≥2500g	0			0			0			0		
birth weight <2500g	-2.432	-2.539; -2.326	*	-2.432	-2.539; -2.326	*	-2.612	-2.712; -2.512	*	-2.606	-2.707; -2.506	*
birth weight <2500g x age	0.068	0.059; 0.076	*	0.070	0.061; 0.078	*	0.067	0.060; 0.074	*	0.068	0.061; 0.076	*
Male sex	0			0			0			0		
Female sex (sex)	-0.086	-0.185; 0.013	0.088	0.036	-0.041; 0.114	0.357	-0.102	-0.200; -0.003	0.044	0.057	-0.018; 0.131	0.136
sex x age	0.016	0.010; 0.022	*	0.014	0.008; 0.020	*	0.016	0.010; 0.021	*	0.012	0.007; 0.017	*
RMMCH	0			0			0			0		
Cohort	0.261	0.066; 0.456	0.009	0.055	-0.10; 0.205	0.475	0.252	0.104; 0.399	0.001	0.037	-0.074; 0.148	0.514
Cohort x age	0.017	0.009; 0.025	*	0.022	0.014; 0.030	*	0.017	0.011; 0.022	*	0.021	0.015; 0.026	*
age (weeks)	0.032	0.029; 0.036	*	0.019	0.011; 0.027	*	0.036	0.034; 0.039	*	0.015	0.011; 0.020	*
parity = 0	0			0			ǂ			ǂ		
parity = 1	0.159	0.029; 0.290	0.017	0.197	0.092; 0.302	*	ǂ			ǂ		
parity = 1 x age	-0.002	-0.010; 0.006	0.700	-0.003	-0.011; 0.005	0.488	ǂ			ǂ		
parity ≥ 2	0.056	-0.075; 0.186	0.404	0.230	0.115; 0.345	*	ǂ			ǂ		
parity ≥ 2 x age	-0.007	-0.015; 0.001	0.085	-0.011	-0.020; -0.002	0.015	ǂ			ǂ		
Any ARVs	0			0			0			0		
No ARVs	-0.383	-0.570; -0.197	*	-0.148	-0.294; -0.001	0.048	-0.440	-0.624; -0.257	*	-0.164	-0.305; -0.024	0.022
No ARVs x age	0.001	-0.011; 0.012	0.906	-0.006	-0.017; 0.006	0.332	-0.010	-0.020; 0.001	0.081	-0.014	-0.024; -0.034	0.009
ARVs missing information	0.084	-0.251; 0.419	0.623	0.089	-0.172; 0.350	0.505	-0.536	-0.840; -0.232	0.001	-0.267	-0.499; -0.036	0.024
ARVs missing information x age	-0.022	-0.041; -0.002	0.03	-0.018	-0.037; 0.001	0.067	-0.002	-0.018; 0.014	0.828	-0.006	-0.022; 0.009	0.436
25–35 years^¶^	0			0			ǂ			ǂ		
Young mother	-0.028	-0.151; 0.096	0.661	0.036	-0.067; 0.140	0.492	ǂ			ǂ		
Young mother x age	-0.0002	-0.008; 0.007	0.959	-0.003	-0.011; 0.005	0.448	ǂ			ǂ		
Older mother	-0.288	-0.419; -0.158	*	-0.119	-0.225; -0.012	0.028	ǂ			ǂ		
Older mother x age	0.006	-0.002; 0.014	0.171	0.004	-0.004; 0.012	0.364	ǂ			ǂ		

¶Maternal age

ǂNot included in the model

*p<0.0001

†Reference categories: Feeding–any breastfeeding, Birth weight - ≥2500g, Sex–males, Cohort–McCord, parity– 0 children

#### Main model (adjusted analysis, including parity)

As expected, infants with a low birth weight (LBW) had a lower birth WAZ compared to normal birth weight infants, but over time, growth was significantly faster among LBW infants (adjusted ß 0.07 per week, p-value for interaction <0.0001). Similarly, infants born to women with parity ≥1 had a higher birth WAZ but experienced a slower increase in WAZ; although the latter effect was small (adjusted ß = -0.011) and only statistically significant for infants of mothers with parity ≥2. In this cohort of HEU infants, females experienced faster growth (adjusted ß 0.014, p-value for interaction <0.0001) compared to male infants.

Although BF infants had a marginally greater birth WAZ compared to infants who received FF (any BF infants p = 0.298), their growth over time was slower compared to infants who received infant formula (and BF infants adjusted ß – 0.012 p-value for interaction = 0.011, missing feeding adjusted ß -0.01 p-value for interaction = 0.173). When including all infants, thus not taking parity into account (Table 3, right side), the effects were similar.

### Sensitivity analyses

This same analysis was conducted among infants with a birth weight ≥2 500g (as a sensitivity analysis) and the direction of these effects remained the same, although the magnitude tended to be smaller (S2 Table). A model including maternal CD4, antiretrovirals and maternal age (S1 Table for all infants and S3 Table for normal-weight infants) showed no significant effect of these on growth over time.

## Discussion

In this cohort of HEU infants, mothers with more advanced disease, and those not on antiretrovirals, delivered infants with a lower birth weight compared to mothers with less advanced disease, and those who received any antiretrovirals, whether as prophylaxis or triple ART. In contrast, in this cohort of mostly formula fed infants, no maternal HIV disease factors were associated with postnatal growth, with postnatal WAZ increasing in all children.

Evidence is mixed for the effect of antiretrovirals on birth weight and preterm deliveries, although triple ART, especially the use of protease inhibitor-containing (PI) regimens, either pre-conception or initiated during pregnancy appears to increase the risk of both LBW and preterm deliveries [27,28].The PROMISE trial found no significant differences between three regimens for both very preterm (<34 weeks) and very low birth weight (<1500g) [29]. We found antiretroviral use protective against LBW. In our study, relatively few women would have conceived on ART and very few were on PI-containing regimens. Maternal triple ART would have been restricted to mothers with advanced disease. In addition, all types of antiretroviral exposure were combined in this analysis so the effects seen are probably due to the partial mitigation of the effects of severe maternal disease by antiretrovirals.

Most HEU infants in the IeDEA-SA collaboration were formula fed—the predominant feeding modality at both sites. MBF infants experienced marginally slower growth compared to FF infants. Although MBF infants experienced slower growth, the effect was very small and it is difficult to draw robust conclusions particularly as less than 10% of infants in this HEU cohort received any breast milk. Furthermore, we did not have detailed data on confounding factors that could impact growth and feeding choice, such as maternal education [4,8,16,17] and socioeconomic status [4,16,17]. Goga et al [14] found differences between sites for the combined end-point of HIV transmission or death, and Ramokolo et al found differences in growth across the same sites [30], thus the effect of different settings and patient characteristics within these settings are an important consideration. It is important to note the reality that most infants were not exclusively breastfed and many were FF. While this may have changed recently with the introduction of option B/B+ in South Africa, strategies to support breastfeeding and optimise growth outcomes in the context of actual infant feeding choices need to be developed and encouraged.

Advanced maternal disease has been shown to negatively impact on growth [4,16], although in this analysis, maternal CD4 and the use of antiretrovirals did not have a significant impact on growth. This may be because maternal antiretroviral use and CD4 data were limited to the antenatal period and may not reflect the maternal disease status after delivery. In addition, the availability of triple therapy for all women with a CD4 cell count <350 cells/μl after 2010 may have mitigated the adverse effects of severe maternal disease seen in studies prior to such antiretroviral availability.

In this analysis, female infants experienced faster growth compared to male infants. Interestingly, previous studies examining growth among HEU infants have mostly not examined the effect of infant sex [5,8,16]. However, Kuhn et al found that female infants had lower actual weight compared to male infants between 1 week and 4 months of age [4], while in a Ugandan study female infants had lower odds of experiencing stunting (length-for-age z-score <-2) or being underweight (WAZ <-2) compared to male infants [17].

### Strengths and limitations

The major strength of this study is the large number of mother-infant dyads included and the description of feeding practices and growth in infants in a routine care setting. Most previous studies of growth in HEU infants in South Africa have been restricted to research cohorts and randomised controlled trial data, where results may differ compared to infants in routine care. However, the reliance on routinely collected data meant that a number of key variables were missing. For example, although only 5% of birth length and head circumference were missing, these were not regularly measured and recorded at follow-up visits so we were unable to examine length-for-age and weight-for-length z-scores over time. Furthermore, although standard practice was employed for growth monitoring, no quality assurance processes were in place. Information on antiretrovirals given to infants themselves for PMTCT was inadequate thus we were unable to take it into account. Similarly, the introduction of solids was not routinely assessed and could not be included in the multivariate analyses. A previous study evaluating infant feeding practices found that 47% of HIV-infected mothers did not initiate breastfeeding; however, 67% of these mothers who initiated FF were practising mixed formula feeding by 3 weeks of age [14]. Mixed FF also comprises mixed feeding; as the introduction of solids was not assessed the extent of mixed FF in this cohort of HEU infants is unknown. Feeding modality and maternal disease severity are known to influence risk of infection in infants [4,10] which would affect growth. However as opportunistic infections were not routinely recorded at sites, this was not investigated.

As only antenatal maternal disease-related factors were included in this analysis, it could account for our finding of an impact on birth WAZ, but not longitudinal WAZ. The impact of postnatal maternal disease severity may have an impact on infant growth outcomes, particularly if infants are breastfed, as it’s been shown that mothers who have more advanced disease have poorer infant outcomes [4,5,8,15–17,31–33], not merely limited to growth

In our cohort of HEU infants birth WAZ was low overall, but whether this was as a result of HIV-exposure or not is unknown. A comparison between HEU infants and unexposed uninfected infants is needed to investigate this further. Two South African studies have found that HEU infants have similar growth outcomes compared to unexposed infants, with one study only finding differences between the ages of 25–39 weeks [5,30].

## Conclusion

Despite the limitations imposed by routine clinical data, this analysis shows that maternal antiretrovirals and less severe maternal disease have a positive impact on birth weight. We also found that any breastfeeding was rare in this cohort with MBF infants experiencing only marginally slower growth compared to FF infants. With the introduction of option B/B+ it will be important to examine the impact on infant feeding pattern, infant morbidity and the impact on growth in the growing population of HEU infants.

## Supporting Information

S1 Table Longitudinal linear regression weight-for-age z-scores 0–28 weeks including parity and maternal CD4 (all infants) (PDF)

S2 Table Longitudinal linear regression weight-for-age z-scores among normal birth weight infants (PDF)

S3 Table Longitudinal linear regression weight-for-age z-scores 0–28 weeks including parity and maternal CD4 (normal birth weight infants) (PDF)
