# Supplementary material for: Growth of HIV-Exposed Uninfected Infants in the First 6 Months of Life in South Africa: The IeDEA-SA Collaboration
Source: PLoS One. 2016 Apr 6;11(4):e0151762. doi: 10.1371/journal.pone.0151762 (PMC4822941; doi:10.1371/journal.pone.0151762)
Supplement: S1 Table — (PDF) [file pone.0151762.s001.pdf]

S1 Table Longitudinal linear regression weight-for-age z-scores 0-28 weeks including parity and maternal CD4 (all infants, n=1387)

	unadjusted			***adjusted***		
variables	**unadjusted ß**	**95% CI**	**p-value**	**adjusted ß**	**95% CI**	**p-value**
formula feeding	0			0		
any breastfeeding	-0.004	-0.205; 0.197	0.969	0.113	-0.048; 0.274	0.169
breastfeeding x age	-0.011	-0.023; 0.001	0.074	-0.010	-0.022; 0.002	0.078
unknown feeding	-0.061	-0.042; 0.299	0.741	0.065	-0.222; 0.353	0.657
unknown feeding x age	-0.004	-0.022; 0.014	0.697	-0.013	-0.031; 0.005	0.159
birth weight ≥2500g	0			0		
birth weight <2500g	-2.303	-2.445; -2.160	*	-2.302	-2.446; -2.158	*
birth weight <2500g x age	0.056	0.043; 0.068	*	0.056	0.043; 0.068	*
age (weeks)	0.032	0.028; 0.037	*	0.025	0.012; 0.038	*
parity = 0	0			0		
parity = 1	0.222	0.062; 0.383	0.006	0.229	0.097; 0.362	0.001
parity = 1 x age	-0.013	-0.024; -0.003	0.016	-0.015	-0.026; -0.004	0.010
parity = 2	0.088	-0.071; 0.248	0.278	0.205	0.062; 0.348	0.005
parity = 2 x age	-0.012	-0.023; -0.001	0.028	-0.013	-0.025; -0.001	0.036
Any ARVs	0			0		
no ARVs	-0.356	-0.652; -0.599	0.018	-0.05	-0.291; 0.190	0.682
no ARVs x age	-0.009	-0.031; 0.013	0.418	-0.012	-0.033; 0.010	0.294
ARVs missing information	-0.182	-0.732; 0.368	0.517	0.021	-0.419; 0.461	0.924
ARVs missing x age	-0.028	-0.059; 0.004	0.083	-0.021	-0.051; 0.010	0.187
CD4 ≥500	0			0		
CD4 <200	-0.317	-0.506; -0.128	0.001	-0.108	-0.261; 0.045	0.168

	unadjusted			***adjusted***		
variables	**unadjusted ß**	**95% CI**	**p-value**	**adjusted ß**	**95% CI**	**p-value**
CD4 <200 x age	0.009	-0.004; 0.022	0.174	0.007	-0.005; 0.020	0.251
200 < CD4 < 500	-0.047	-0.190; 0.096	0.518	0.040	-0.075; 0.155	0.491
200 < CD4 < 500 x age	0.006	-0.004; 0.016	0.234	0.003	-0.006; 0.013	0.498
Male sex	0			0		
Female sex (sex)	-0.160	-0.282; -0.039	0.010	0.009	-0.089; 0.108	0.854
sex x age	0.016	0.008; 0.024	*	0.013	0.005; 0.021	0.002
RMMCH	0			0.000		
Cohort	0.044	-0.182; 0.270	0.702	-0.061	-0.239; 0.118	0.506
Cohort x age	0.026	0.016; 0.036	*	0.029	0.018; 0.039	*
25-35 years¶	0			0		
young mother	-0.026	-0.181; 0.130	0.746	-0.018	-0.152; 0.116	0.792
young mother x age	-0.003	-0.014; 0.008	0.593	-0.006	-0.018; 0.005	0.260
older mother	-0.233	-0.395; -0.072	0.005	-0.101	-0.236; 0.032	0.139
older mother x age	-0.002	-0.013; 0.009	0.749	-0.004	-0.014; 0.007	0.511

*p<0.0001
