# Supplementary material for: Growth of HIV-Exposed Uninfected Infants in the First 6 Months of Life in South Africa: The IeDEA-SA Collaboration
Source: PLoS One. 2016 Apr 6;11(4):e0151762. doi: 10.1371/journal.pone.0151762 (PMC4822941; doi:10.1371/journal.pone.0151762)
Supplement: S2 Table — (PDF) [file pone.0151762.s002.pdf]

S2 Table Longitudinal linear regression, weight-for-age z-scores among normal birth weight infants

	Model including parity ; n=1953						***Model including all infants; n=2171***					
	unadjusted			***adjusted***			***unadjusted***			***adjusted***		
variables	**unadjusted ß**	**95% CI**	**p-value**	**adjusted ß**	**95% CI**	**p-value**	**unadjusted ß**	**95% CI**	**p-value**	**adjusted ß**	**95% CI**	**p-valu**
formula feeding	0			0			0			0		
any breastfeeding	0.012	-0.130; 0.155	0.865	-0.005	-0.147; 0.137	0.946	0.022	-0.114; 0.0158	0.753	-0.006	-0.142; 0.130	0.934
breastfeeding x age	-0.009	-0.019; 0.001	0.095	-0.004	-0.014; 0.006	0.425	-0.010	-0.019; -0.008	0.033	-0.004	-0.013; 0.005	0.404
unknown feeding	-0.112	-0.382; 0.158	0.415	-0.108	-0.378; 0.161	0.43	-0.040	-0.297; 0.216	0.757	-0.013	-0.027; 0.001	0.061
unknown feeding x age	-0.002	-0.017; 0.013	0.768	-0.012	-0.027; 0.003	0.112	-0.007	-0.021; 0.007	0.313	-0.042	-0.297; 0.213	0.648
age (weeks)	0.022	0.019; 0.026	*	0.017	0.008; 0.026	*	0.025	0.023; 0.028	*	0.013	0.008; 0.017	*
Male sex	0			0			0			0		
Female sex (sex)	0.040	-0.041; 0.121	0.336	0.026	-0.055; 0.107	0.524	0.043	-0.033; 0.120	0.267	0.041	-0.036; 0.117	0.295
sex x age	0.015	0.008; 0.021	*	0.017	0.010; 0.023	*	0.011	0.006; 0.017	*	0.013	0.007; 0.018	*
RMMCH	0			0			0			0		
Cohort	0.004	-0.144; 0.152	0.954	0.042	-0.108; 0.192	0.582	0.019	-0.089; 0.127	0.728	0.0247	-0.084; 0.133	0.654
Cohort x age	0.029	0.021; 0.037	*	0.028	0.019; 0.036	*	0.026	0.020; 0.032	*	0.0251	0.019; 0.031	*
parity = 0	0			0			ǂ			ǂ		
parity = 1	0.160	0.054; 0.266	0.003	0.176	0.067; 0.285	0.002	ǂ			ǂ		
parity = 1 x age	-0.005	-0.013; 0.004	0.261	-0.004	-0.012; 0.005	0.411	ǂ			ǂ		
parity ≥ 2	0.187	0.080; 0.294	0.001	0.240	0.120; 0.361	*	ǂ			ǂ		
parity ≥ 2 x age	-0.015	-0.023; -0.006	0.001	-0.013	-0.023; -0.004	0.006	ǂ			ǂ		

	Model including parity ; n=1953						***Model including all infants; n=2171***					
	unadjusted			***adjusted***			***unadjusted***			***adjusted***		
variables	**unadjusted ß**	**95% CI**	**p-value**	**adjusted ß**	**95% CI**	**p-value**	**unadjusted ß**	**95% CI**	**p-value**	**adjusted ß**	**95% CI**	**p-valu**
25 - 35 years[¶]	0			0			ǂ			ǂ		
Young mother	-0.047	-0.148; 0.054	0.365	0.032	-0.076; 0.139	0.564	ǂ			ǂ		
Young mother x age	0.0003	-0.008; 0.008	0.941	-0.004	-0.013; 0.004	0.345	ǂ			ǂ		
Older mother	-0.126	-0.238; -0.015	0.026	-0.175	-0.290; -0.061	0.003	ǂ			ǂ		
Older mother x age	0.001	-0.007; 0.010	0.759	0.007	-0.002; 0.016	0.141	ǂ			ǂ		
Any ARVs	0			0			0			0		
No ARVs	-0.055	-0.217; 0.108	0.509	-0.053	-0.214; 0.109	0.523	-0.045	-0.197; 0.108	0.565	-0.053	-0.204; 0.099	0.496
No ARVs x age	-0.011	-0.024; 0.002	0.092	-0.011	-0.024; 0.002	0.098	-0.016	-0.028; -0.004	0.009	-0.013	-0.025; -0.001	0.029
ARVs missing information	0.218	-0.058; 0.495	0.122	0.201	-0.072; 0.475	0.15	0.153	-0.101; 0.407	0.239	0.113	-0.139; 0.365	0.381
ARVs missing information x age	-0.022	-0.043; -0.002	0.033	-0.016	-0.037; 0.004	0.111	-0.030	-0.049; -0.011	0.002	-0.021	-0.039; -0.002	0.029

*p<0.0001 ǂNot included in the model
