# Supplementary material for: Growth of HIV-Exposed Uninfected Infants in the First 6 Months of Life in South Africa: The IeDEA-SA Collaboration
Source: PLoS One. 2016 Apr 6;11(4):e0151762. doi: 10.1371/journal.pone.0151762 (PMC4822941; doi:10.1371/journal.pone.0151762)
Supplement: S3 Table — (PDF) [file pone.0151762.s003.pdf]

S3 Table Longitudinal linear regression weight-for-age z-scores 0-28 weeks including parity and maternal CD4 (normal birth weight infants, n = 1202)

| | ***unadjusted*** | | | ***adjusted*** | | |
|---|---|---|---|---|---|---|
| ***variables*** | **unadjusted ß** | **95% CI** | **p-value** | **adjusted ß** | **95% CI** | **p-value** |
| formula feeding | 0 | | | 0 | | |
| any breastfeeding | 0.068 | -0.107; 0.244 | 0.446 | 0.063 | -0.113; 0.239 | 0.483 |
| breastfeeding x age | -0.007 | -0.020; 0.006 | 0.268 | -0.001 | -0.015; 0.012 | 0.823 |
| unknown feeding | -0.077 | -0.388; 0.233 | 0.625 | -0.021 | -0.332; 0.291 | 0.895 |
| unknown feeding x age | -0.002 | -0.020; 0.017 | 0.871 | -0.017 | -0.036; 0.002 | 0.075 |
| | | | | | | |
| birth weight ≥2500g | ǂ | | | ǂ | | |
| birth weight <2500g | ǂ | | | ǂ | | |
| birth weight <2500g x age | ǂ | | | ǂ | | |
| | | | | | | |
| age (weeks) | 0.025 | 0.021; 0.030 | * | 0.022 | 0.008; 0.036 | 0.002 |
| | | | | | | |
| parity = 0 | 0 | | | 0 | | |
| parity = 1 | 0.232 | 0.096; 0.368 | 0.001 | 0.237 | 0.097; 0.376 | 0.001 |
| parity = 1 x age | -0.017 | -0.028; -0.006 | 0.003 | -0.017 | -0.029; -0.005 | 0.005 |
| parity = 2 | 0.201 | 0.064; 0.338 | 0.004 | 0.021 | 0.061; 0.367 | 0.006 |
| parity = 2 x age | -0.018 | -0.029; -0.006 | 0.002 | -0.014 | -0.027; -0.011 | 0.033 |
| | | | | | | |
| Any ARVs | 0 | | | 0 | | |
| no ARVs | 0.024 | -0.253; 0.300 | 0.866 | 0.014 | -0.261; 0.290 | 0.919 |
| no ARVs x age | -0.032 | -0.058; -0.006 | 0.014 | -0.028 | -0.054; -0.002 | 0.035 |
| ARVs missing information | 0.270 | -0.228; 0.767 | 0.288 | 0.224 | -0.270; 0.718 | 0.374 |
| ARVs missing x age | -0.033 | -0.065; -0.0002 | 0.049 | -0.022 | -0.055; 0.010 | 0.183 |

| | *unadjusted* | | | *adjusted* | | |
|---|---|---|---|---|---|---|
| ***variables*** | **unadjusted ß** | **95% CI** | **p-value** | **adjusted ß** | **95% CI** | **p-value** |
| CD4 ≥500 | 0 | | | 0 | | |
| CD4 <200 | -0.141 | -0.306; 0.023 | 0.093 | -0.162 | -0.324; 0.004 | 0.056 |
| CD4 <200 x age | 0.009 | -0.005; 0.023 | 0.197 | 0.013 | -0.001; 0.027 | 0.066 |
| 200 < CD4 < 500 | 0.013 | -0.108; 0.133 | 0.831 | 0.009 | -0.111; 0.130 | 0.882 |
| 200 < CD4 < 500 x age | 0.004 | -0.006; 0.014 | 0.399 | 0.004 | -0.006; 0.014 | 0.407 |
| | | | | | | |
| Male sex | 0 | | | 0 | | |
| Female sex (sex) | 0.010 | -0.095; 0.114 | 0.856 | -0.003 | -0.107; 0.101 | 0.954 |
| sex x age | 0.013 | 0.004; 0.022 | 0.004 | 0.015 | 0.006; 0.024 | 0.001 |
| | | | | | | |
| RMMCH | 0 | | | 0 | | |
| Cohort | -0.084 | -0.266; 0.099 | 0.368 | -0.067 | -0.251; 0.118 | 0.480 |
| Cohort x age | 0.035 | 0.025; 0.046 | * | 0.036 | 0.025; 0.047 | * |
| | | | | | | |
| 25-35 years[¶] | 0 | | | 0 | | |
| young mother | -0.087 | -0.218; 0.045 | 0.197 | -0.018 | -0.159; 0.123 | 0.804 |
| young mother x age | -0.002 | -0.013; 0.009 | 0.759 | -0.008 | -0.020; 0.004 | 0.207 |
| older mother | -0.111 | -0.253; 0.031 | 0.127 | -0.141 | -0.288; 0.005 | 0.058 |
| older mother x age | -0.005 | -0.017; 0.006 | 0.365 | -0.002 | -0.014; 0.010 | 0.715 |

*p<0.0001 ǂNot included in the model
